# An Approach to Elucidate NBS1 Function in DNA Repair Using Frequent Nonsynonymous Polymorphism in Wild Medaka (*Oryzias latipes*) Populations

**DOI:** 10.1371/journal.pone.0170006

**Published:** 2017-01-20

**Authors:** Kento Igarashi, Junya Kobayashi, Takafumi Katsumura, Yusuke Urushihara, Kyohei Hida, Tomomi Watanabe-Asaka, Hiroki Oota, Shoji Oda, Hiroshi Mitani

**Affiliations:** 1 Department of Integrated Biosciences, Graduate School of Frontier Sciences, The University of Tokyo, Kashiwa, Japan; 2 Department of Genome Repair Dynamics, Radiation Biology Center, Kyoto University, Kyoto, Japan; 3 Department of Anatomy, Kitasato University School of Medicine, Sagamihara, Japan; Chang Gung University, TAIWAN

## Abstract

*Nbs1* is one of the genes responsible for Nijmegen breakage syndrome, which is marked with high radiosensitivity. In human NBS1 (hNBS1), Q185E polymorphism is known as the factor to cancer risks, although its DSB repair defect has not been addressed. Here we investigated the genetic variations in medaka (*Oryzias latipes*) wild populations, and found 40 nonsynonymous single nucleotide polymorphisms (SNPs) in medaka *nbs1* (*olnbs1*) gene within 5 inbred strains. A mutation to histidine in Q170 residue in olNbs1, which corresponds to Q185 residue of hNBS1, was widely distributed in the closed colonies derived from the eastern Korean population of medaka. Overexpression of H170 type olNbs1 in medaka cultured cell lines resulted in the increased accumulation of olNbs1 at laser-induced DSB sites. Autophosphorylation of DNA-dependent protein kinase at T2609 was suppressed after the γ-ray irradiation, which was followed by prolonged formation of γ-H2AX foci and delayed DSB repair. These findings suggested that the nonsynonymous SNP (Q170H) in *olnbs1*, which induced DSB repair defects, is specifically distributed in the eastern Korean population of medaka. Furthermore, examination using the variation within wild populations might provide a novel method to characterize a driving force to spread the disease risk alleles.

## Introduction

Homologous recombination (HR) and nonhomologous end joining (NHEJ) are the two main pathways in DNA double strand break (DSB) repair. NBS1 is a component of Mre11–Rad50–Nbs1 (MRN) complex which plays an essential role in HR pathway in higher eukaryotes [1] and competes for the binding to DSB sites with DNA-dependent protein kinase (DNA-PK), a major component in NHEJ pathway. NBS1 has a forkhead associated (FHA) domain and two brca1-carboxy termini domains (BRCT1 and BRCT2) in its N-terminal region and accumulates at DSB sites by interactions between the BRCT domains and phosphorylated histone H2AX (γ-H2AX) [2].

Alleles that increase cancer risk has been investigated for decades. Recent human genome analysis are highlighting the functional significance of SNPs in human diseases [3–5]. Human NBS1 (hNBS1) is also a target of studies on the relationship between mutations and cancer risks [6]. I171V is a rare SNP which is firstly identified in a patient of acute leukemia in Japanese and its heterozygote can increase breast cancer risk [7–9]. I171V mutation locates in the BRCT domain of hNBS1 and decreases formation of γ-H2AX foci in Nijmegen breakage syndrome (NBS) cells [8]. Q185E is a widely prevailed polymorphism in human (e. g. Q185 allele frequency is 44.7% in Tokyo) [9,10]. A variety of previous studies reported that Q185 allele is associated with higher cancer risks, such as bladder cancer in Swedish people [11] and acute myeloid leukemia in Chinese [12], suggesting that the mutation to glutamic acid at Q185 residue in hNBS1 has some biological significance. Nonetheless, it remains unclear how a mutation at Q185 residue impacts the molecular function of NBS1 and DSB repair.

Medaka (*Oryzias latipes*) is a small fresh-water fish that is widely spread throughout East Asia [13,14] and is used as a model in radiation biology. Impaired DSB repair was previously elucidated in the cells derived from a radiation-sensitive strain of medaka using immunofluorescence staining of γ-H2AX and phosphorylated DNA-PK at T2609 [15]. Recently, there are several studies using medaka to analyze the functional differences among genetic polymorphisms for understanding those in humans [16–18]. Since medaka has highly differentiated geographical populations with large genetic diversity [19], the similar genetic polymorphisms equivalent to humans are occasionally found in the wild populations of medaka. Therefore, medaka has been described as “a natural library” of genetic variation in humans for orthologous genes [16]. Several labs in the universities and institutes maintain wild medaka as lab-stocks for studies on functional differences of genetic polymorphisms. In the present study, we first found 40 nonsynonymous polymorphisms in the amino acid sequence of medaka NBS1 gene (*olnbs1*) amino acid sequence within 5 inbred strains (Hd-rR, HNI, Kaga, HSOK, and Nilan) using the sequence information provided by the National BioResource Project (NBRP) medaka SNP browser. Among the 40 nonsynonymous polymorphisms, Q170H mutation was found only in HSOK inbred strain from the subpopulation habitat in the eastern part of Korea, and this Q170 in olNbs1 corresponds to Q185 in hNBS1 by amino acid sequence alignment between hNBS1 and olNbs1 (from Hd-rR strain: ENSORLG00000009450.1). The distribution of Q170H polymorphism was dominant in the eastern Korean group by the sequence analysis of the medaka lab-stocks from wild populations. To elucidate DSB repair function of olNbs1 (H170), we also conducted a neutral comet assay, laser micro-irradiation and examination of γ-H2AX and DNA-PK catalytic subunit phosphorylated Thr2609 (DNA-PKcs pT2609) foci formation by using the cell lines overexpressing olNbs1-Venus or olNbs1 (H170)-Venus. Our results demonstrated that olNbs1 (H170), which is predominantly distributed in the eastern Korean group, caused delay of DSB repair in cultured cells.

## Results

### olNbs1 is polymorphic in 5 inbred strains of medaka

**Fig 1:**
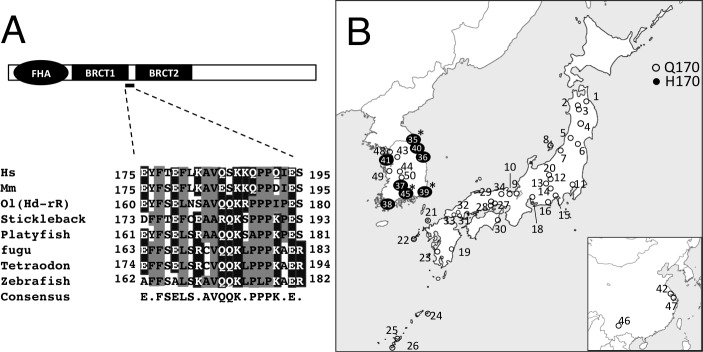
Conserved sequences around Q170 residue in olNbs1 and distribution of Q170/H170 alleles in wild medaka populations. (A) Schematic drawing of NBS1 protein domain structure (top) and alignment of amino acid sequences around Q170 residue of olNbs1 (bottom) are shown. FHA domain and two BRCT domains are present in the N-terminal region of NBS1 and Q170 locates in the flange part between BRCT1 and BRCT2 domains in olNbs1. Twenty-one amino acids for Hs, *Homo sapiens* (from E175 to S195 of ENST00000265433.7); Mm, *Mus musculus* (from E175 to S195 of ENSMUST00000029879.14); Ol, *Oryzias latipes* (Hd-rR, from E160 to S180 of ENSORLG00000009450.1); stickleback (from D173 to S193 of ENSGACT00000016251); platyfish (from E161 to S181 of ENSXMAT00000007002); fugu (from E163 to R183 of ENSTRUT00000005862); tetraodon (from E174 to R194 of ENSTNIT00000021752), zebrafish (from A162 to R182 of ENSDART00000058974) are aligned from the ENSEMBL database and the consensus amino acids are indicated. Hydrophobic residues are highlighted in gray boxes, and amino acid residues with a long straight side chain (≥ 3 carbons) are highlighted in black boxes. (B) Distribution of olNbs1 (Q170) and olNbs1 (H170) alleles in the wild medaka populations. Parenthesized numbers refer to the ID numbers listed in Table 1. Open circles represent the collection sites where homozygotes of the olNbs1 (Q170) allele were found. Filled circles with numbers represent the collection sites where homozygotes of the olNbs1 (H170) alleles were found. Asterisks indicate the collection sites where heterozygotes of the olNbs1 (Q170) allele and the olNbs1 (H170) allele were found.

First we attempted to identify candidate nonsynonymous polymorphisms in *olnbs1* within wild medaka populations, which can lead to functional impacts on DSB repair. We found 40 nonsynonymous polymorphisms in the *olnbs1* genomic DNA sequence of the 5 inbred strains (Hd-rR, HNI, Kaga, HSOK, and Nilan) in the sequence information provided by the NBRP medaka (S1 Table). Among these, Q170H mutation in olNbs1 was specifically appeared in HSOK and of great interest because the amino acid sequence alignment between hNBS1 and olNbs1 indicates that Q170 in olNbs1 corresponds to Q185 in hNBS1 (S1 Fig). Furthermore, Q170H in olNbs1 and Q185E in hNBS1 mutations are predicted to locate in the flange part between the BRCT1 and BRCT2 domains (Fig 1A and S1 Fig). Only Q170H mutation in *olnbs1* is located in the flange part between the BRCT1 and BRCT2 domains within the 40 nonsynonymous polymorphisms in *olnbs1* (S1 Table). The local amino acid residues around Q170H mutation in olNbs1 are strongly conserved among animal species (Fig 1A), which is composed of 6–7 hydrophobic residues and 7–8 residues with a long straight side chain with more than 3 carbons (K, R, Q, and E). Histidine in these conserved residues was found only in H170 type olNbs1 even though histidine is a basic amino acid like the others (K and R), suggesting that Q170H mutation has a marked impact on Nbs1 functions.

**Table 1:** The list of lab-stocks of wild medaka used in this study.

No.	Place name	N	Q or H	Group	Provider	Primers (F—R)	Accession No.
1	Kamikita	1	Q/Q	N.JPN [20]	UT	60–33	LC159330
2	Hirosaki	1	Q/Q	N.JPN [20]	UT	60–33	LC159331
3	Oodate	1	Q/Q	N.JPN [20]	UT	60–33	LC159332
4	Yokote	1	Q/Q	N.JPN [20]	UT	60–33	LC159333
5	Tsuruoka	1	Q/Q	N.JPN [20]	UT	60–33	LC159334
6	Yamagata	1	Q/Q	N.JPN [20]	UT	60–33	LC159335
7	Niigata	1	Q/Q	N.JPN [20]	UT	60–33	LC159336
8	Ryotsu	1	Q/Q	N.JPN [20]	UT	60–33	LC159337
9	Maizuru	1	Q/Q	N.JPN [20]	UT	60–33	LC159338
10	Miyazu	1	Q/Q	N.JPN [20]	UT	60–33	LC159339
11	Tokyo	1	Q/Q	S.JPN [20]	UT	60–33	LC159340
12	Toyota	1	Q/Q	S.JPN [20]	UT	60–33	LC159341
13	Oomachi	1	Q/Q	S.JPN [20]	UT	60–33	LC159342
14	Suwa	1	Q/Q	S.JPN [20]	UT	60–33	LC159343
15	Shizuoka	1	Q/Q	S.JPN [20]	UT	60–33	LC159344
16	Iwata	1	Q/Q	S.JPN [20]	UT	60–33	LC159345
18	Nagashima	1	Q/Q	S.JPN [20]	UT	60–33	LC159346
19	Kusu	1	Q/Q	S.JPN [20]	UT	60–33	LC159347
20	Okaya	1	Q/Q	S.JPN [20]	UT	60–33	LC159348
21	Ashibe	1	Q/Q	S.JPN [20]	UT	60–33	LC159349
22	Fukue	1	Q/Q	S.JPN [20]	UT	60–33	LC159350
23	Hiwaki	1	Q/Q	S.JPN [20]	UT	60–33	LC159351
24	Kikai	1	Q/Q	S.JPN [20]	UT	60–33	LC159352
25	Nago	1	Q/Q	S.JPN [20]	UT	60–33	LC159353
26	Gushikami	1	Q/Q	S.JPN [20]	UT	60–33	LC159354
27	Oku	1	Q/Q	S.JPN [20]	UT	60–33	LC159355
28	Okayama	1	Q/Q	S.JPN [20]	UT	60–33	LC159356
29	Kayou	1	Q/Q	S.JPN [20]	UT	60–33	LC159357
30	Tokushima	1	Q/Q	S.JPN [20]	UT	60–33	LC159358
31	Kudamatsu	1	Q/Q	S.JPN [20]	UT	60–33	LC159359
32	Yamaguchi	1	Q/Q	S.JPN [20]	UT	60–33	LC159360
33	Sanyou	1	Q/Q	S.JPN [20]	UT	60–33	LC159361
34	Kasumi	1	Q/Q	S.JPN [20]	UT	60–33	LC159362
35	Yongcheon	2	H/H, H/Q	E.KOR [21]	UT	60–33	LC159363, LC159364, LC159367
36	Sacheon	2	H/H, H/H	E.KOR [21]	UT	60–33	LC159365, LC159368
37	Sinpyeong	2	H/H, H/H	E.KOR [21]	UT	60–33	LC159366, LC159369
38	Jindo	2	H/H, H/H	E.KOR [21]	NBRP	157–158	LC159370, LC159371
39	Geoje	2	H/H, H/Q	E.KOR [21]	NBRP	60–33	LC159374, LC159375
40	Sokcho	2	H/H, H/H	E.KOR [21]	NBRP	60–33	LC159376, LC159377
*41	Daebu	2	H/H, H/H	W.KOR/Chinese [21]	NBRP	60–33	LC159372, LC159373
42	Shanghai	1	Q/Q	W.KOR/Chinese [21]	UT	60–33	LC159378
43	Maegok	1	Q/Q	W.KOR/Chinese [13]	UT	60–33	LC159379, LC159380
44	Bugang	1	Q/Q	W.KOR/Chinese [21]	UT	60–33	LC159381, LC159382
45	Gwangui	1	H/Q	W.KOR/Chinese [21]	NBRP	60–33	LC159383
46	Kunming	1	Q/Q	W.KOR/Chinese [21]	NBRP	60–33	LC159384
47	Xuanlan	1	Q/Q	W.KOR/Chinese [21]	NBRP	60–33	LC159385
48	Samsan	1	Q/Q	W.KOR/Chinese [21]	NBRP	60–33	LC159386
49	Guhang	1	Q/Q	W.KOR/Chinese [21]	NBRP	60–33	LC159387
50	Simcheon	1	Q/Q	W.KOR/Chinese [21]	NBRP	60–33	LC159388, LC159389
	*O*. *luzonensis*	1	Q/Q	out-group	NBRP	157–158	LC159390
	*O*. *curvinotus*	1	Q/Q	out-group	NBRP	157–158	LC159391

ID Number of collection sites, Place name of collection sites, sample sizes (N), and genotype of olNbs1 allele, geographical groups to which the local population belongs determined based on phylogenic analysis using mitotype [13,20,21], provider, primers used and accession number are listed. Daebu (No. 41) samples were classified into the W.KOR group based on the geographical location of its collection site, although the Daebu population has the mitotype of the E.KOR group. UT, The University of Tokyo; NBRP, National BioResource Project.

### High genetic differentiation of olNbs1 alleles among medaka geographical groups

We examined the distribution of both alleles (Q170 and H170) in local medaka populations to clarify whether olNbs1 Q170H amino acid change is a dominant polymorphism in the E.KOR group. 326 bp partial *olnbs1* sequences from exon 4 to intron 5 excluding in/dels were obtained from 116 sequences of 58 wild medaka lab-stocks and the H170 allele was specifically found in the E.KOR group (Table 1 and Fig 1B). *G*_ST_ value calculated based on exon 5 haplotypes, in which the H170 allele is located, was higher than *G*_ST_ values of exon 4 and intron 4 (S2 Fig). In the phylogenetic network using the sequences of *olnbs1* exon 5, we found two major haplotypes: one was the H_6 haplotype in which the H170 allele was located, and the other was H_1 haplotype in which the Q170 allele was located (S3 Fig). Unlike the H170 allele, the Q170 allele was shared among all of the geographical groups and had 9 different haplotypes. The H_9 and H_10 haplotypes obtained from two sister *Oryzias* species (*O*. *curvinotus* and *O*. *luzonensis*) were connected with the H_1 haplotype by a branch, indicating that the H_1 haplotype was close to the common ancestor of *olnbs1* in *O*. *latipes*. Tajima’s *D* was calculated based on 326 bp nucleotide sequences and showed the lowest value, –1.65, in the E.KOR group (0.1 > *p* > 0.05; S4 Fig). A significant negative value of Tajima’s *D* generally represents positive selection. Therefore, these population genetic analyses strongly suggest that the H170 allele has been derived from the Q170 allele, and the frequency of the H170 allele was elevated in the environment of the E.KOR group.

### Deficient DSB repair in cells overexpressing olNbs1 (H170)-Venus

**Fig 2:**
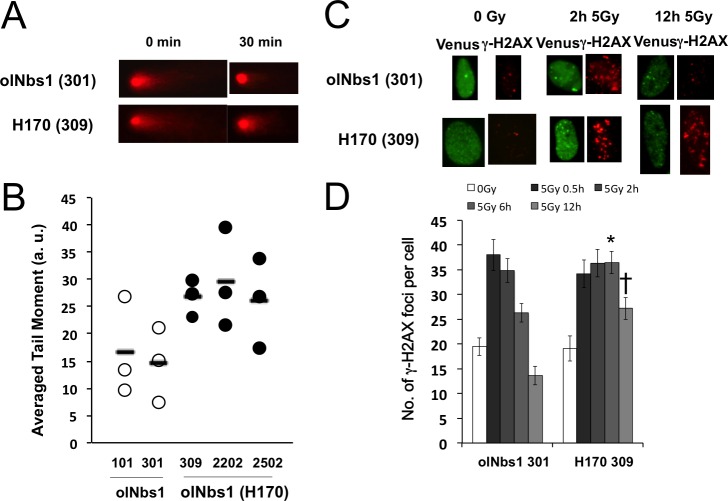
Delayed DSB repair in cells overexpressing olNbs1 (H170)-Venus protein. (A) Typical photographs of electrophoresed nuclei of the cells overexpressing olNbs1-Venus (No. 301) and the cells overexpressing olNbs1 (H170)-Venus (No. 309) are shown. Immediately after the γ-rays irradiation (5 Gy) (0 min), electrophoresed nuclei of both No. 301 cell and No. 309 cell showed comet tailing, indicating that DSB were induced by irradiation. Electrophoresed nuclei of No.309 cell still showed comet tailing 30 min after the irradiation, indicating that DSBs remained not repaired, while DSBs in the nuclei of No.301 cell were repaired within 30 min after the irradiation. (B) Tail moment scores were measured 30 min after the γ-ray irradiation (5 Gy) in the cells overexpressing olNbs1-Venus (Nos. 101 and 301) and olNbs1 (H170)-Venus (Nos. 309, 2202 and 2502). Open and filled circles represent average tail moment scores of at least 100 cells of No.101, 301 cells and No.309, 2202 and 2505 cells, respectively. The experiments were repeated three times and averages of the tail moment scores from triplicate experiments are shown in bars. (C) The cells overexpressing olNbs1-Venus (No. 301) and the cells overexpressing olNbs1 (H170)-Venus (No. 309) were irradiated with γ-rays (5 Gy). Induced γ-H2AX and olNbs1 foci were visualized 2 and 12 h after the irradiation by immunostaining. Foci of γ-H2AX and olNbs1 in the not-irradiated cells are also shown (0 Gy). (D) γ-H2AX foci were counted 30 min, 2, 6 and 12 h after the γ-irradiation (5 Gy) in the cells (No. 301) overexpressing olNbs1-Venus and in the cells (No. 309) overexpressing olNbs1 (H170)-Venus. Data were obtained from one representative experiment, and error bars represent standard errors of the means per cell (n > 20 cells). Statistical analyses were performed using a Student *t* test and * and † represent significant differences between No. 301 cell and No. 309 cell (*p* < 0.005 and *p* < 0.001, respectively).

To clarify the impact of Q170H amino acid change on NBS1 function in DSB repair, we performed a neutral comet assay and examined γ-H2AX foci formation on the olNbs1 (H170)-Venus overexpressing cells. Electrophoretic images of the nuclei and tail moment scores in a neutral comet assay were almost the same between the cell lines expressing olNbs1-Venus and the cell lines expressing olNbs1 (H170)-Venus immediately after the γ-ray irradiation (5 Gy), indicating that the same amounts of DSB were induced in the both cells by the irradiation. Thirty minutes after the irradiation, electrophoretic images of the nuclei of the cells expressing olNbs1-Venus (Nos. 101 and 301) showed short tailing, and their tail moments were about 15 arbitrary units (a.u., Fig 2A and 2B), indicating that DSBs were almost completely repaired. By contrast, the electrophoresed nuclei of the cells expressing olNbs1 (H170)-Venus (Nos. 309, 2202 and 2502) still showed clear tailing, and their tail moments were about 30 a.u. (Fig 2A and 2B), clearly demonstrating that overexpression of olNbs1 (H170) interfered with DSB repair in cultured medaka cells.

The number of γ-H2AX foci increased 30 min after the irradiation in both of the cells overexpressing olNbs1 Q170 and H170 type alleles (Fig 2C and 2D). In the cells expressing olNbs1-Venus, the number of γ-H2AX foci significantly decreased within 6 h after the irradiation and recovered to the control level within 12 h after the irradiation. By contrast, the increased number of γ-H2AX foci was remained for 6 h after the irradiation at the same level as 30 min after the irradiation in the cells overexpressing olNbs1 (H170)-Venus, and slightly decreased 12 h after the irradiation (Fig 2D). This finding strongly suggests that DSBs are recognized and γ-H2AX foci are formed in the both cells overexpressing olNbs1 (H170)-Venus and wild-type olNbs1-Venus, however, DSB repair is delayed and DSBs remain unrepaired for longer than 6 h in the cells overexpressing olNbs1 (H170)-Venus.

### Suppressed autophosphorylation of DNA-PKcs at T2609 resulting from olNbs1 (H170) expression

**Fig 3:**
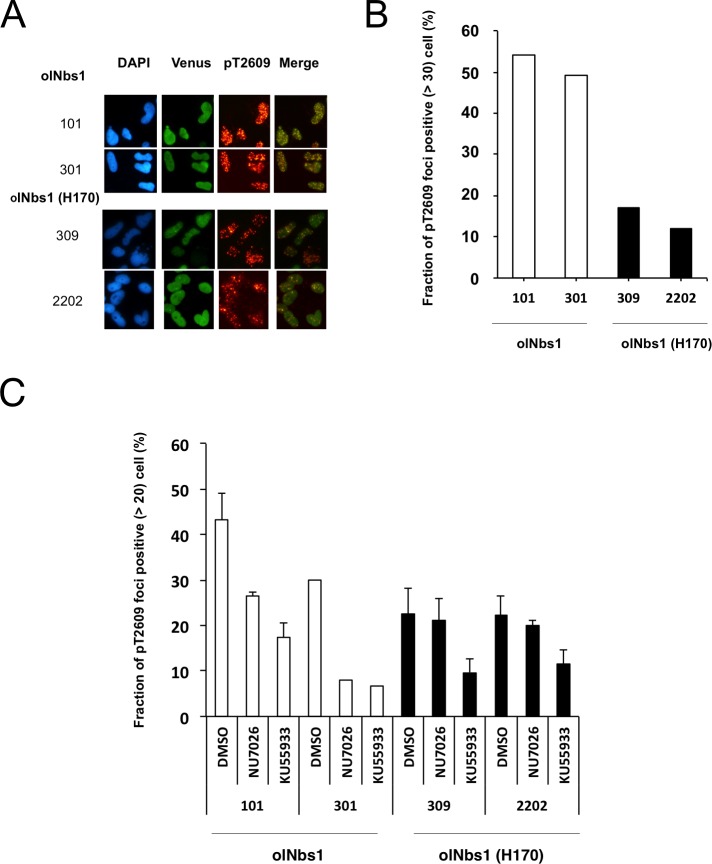
Suppressed autophosphorylation of DNA-PKcs (pT2609) in cells expressing olNbs1 (H170)-Venus protein. (A) The cells overexpressing olNbs1-Venus (Nos. 101 and 301) and the cells overexpressing olNbs1 (H170)-Venus (Nos. 309 and 2202) were stained with DAPI and immunostained with anti-phosphorylated DNA-PKcs (pT2609) antibody 30 min after the γ-ray irradiation (5 Gy). Fluorescent images of DAPI-stained nuclei, expressed Venus-fusion proteins, foci of phosphorylated DNA-PKcs (pT2609) and merged images of the Venus-fusion proteins and phosphorylated DNA-PKcs (pT2609) are shown. (B) Fractions of pT2609 foci positive cells (>30 foci per cell) in the cells overexpressing olNbs1-Venus (No. 101, n = 48; No. 301, n = 65) and in the cells overexpressing olNbs1 (H170)-Venus (No. 309, n = 41; No. 2202, n = 50) are shown. (C) Fractions of pT2609 foci positive cells (>20 foci per cell) in the cells overexpressing olNbs1-Venus (Nos. 101 and 301) and the cells overexpressing olNbs1 (H170)-Venus (Nos. 309 and 2202) treated with DNA-PK inhibitor (NU7026) or ATM inhibitor (KU55933) were counted 30 min after the γ-ray irradiation (5 Gy). At least 40 cells were counted in each experiment and bars represent standard errors of 3 independent experiments.

Phosphorylation of DNA-PKcs at T2609, which induces dissociation of DNA-PK and other repair factors including both NHEJ and HR repair factors from DSB sites [22], was then examined. In the cell lines overexpressing olNbs1-Venus or olNbs1 (H170)-Venus, foci of phosphorylated DNA-PKcs were observed in the nucleus 30 min after the γ-ray irradiation (5 Gy), suggesting that DNA-PKcs are phosphorylated at T2609 (pT2609) in all of the cell lines expressing olNbs1 (Nos. 101 and 301) and olNbs1 (H170) (Nos. 309 and 2202) (Fig 3A). The strong pT2609 signals (more than 30 foci per cell) were observed in about 50% of the cells overexpressing wild-type olNbs1. In contrast, less than 15% of the cells overexpressing olNbs1 (H170) showed pT2609 signals, suggesting that defects in the phosphorylation of DNA-PKcs at T2609 were defective in the cells overexpressing olNbs1 (H170) (Fig 3A and 3B).

Since it is reported that ATM and DNA-PKcs are related in the phosphorylation of DNA-PKcs at T2609, we then evaluate the phosphorylation of DNA-PKcs at T2609 under the existence of ATM inhibitor (KU55933) or DNA-PK inhibitor (NU7026) in the olNbs1-Venus overexpressed cells. In the cells overexpressing olNbs1 (H170)-Venus, pT2609 foci-positive cells were decreased in the existence of KU55933 (Fig 3C). In contrast, pT2609 foci-positive cells did not decrease after NU7026 treatment. These results suggested that phosphorylation of DNA-PKcs by ATM was not inhibited but that autophosphorylation of DNA-PKcs by DNA-PK itself was suppressed in the cells overexpressing olNbs1 (H170)-Venus.

### Prolonged retention of olNbs1 (H170)-venus at DSBs

**Fig 4:**
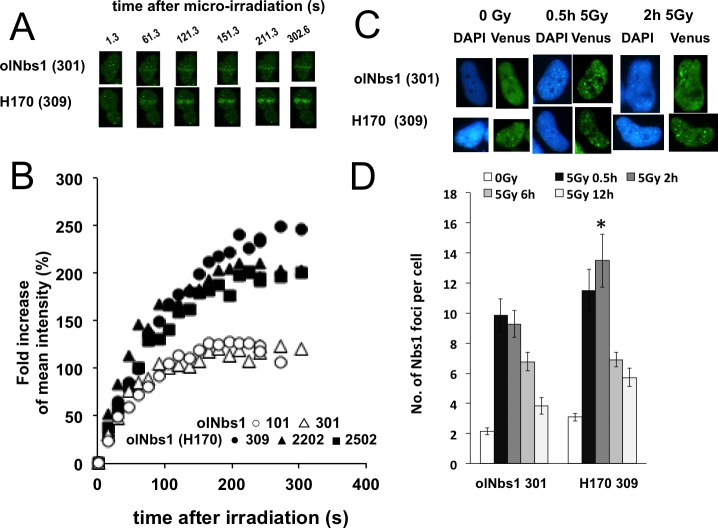
Accumulation of olNbs1-Venus proteins at DSBs. (A) Nuclei of the cells overexpressing olNbs1-Venus (No. 301) and the cells overexpressing olNbs1 (H170)-Venus (No. 309) were micro-irradiated with 405 nm laser and accumulation of olNbs1-Venus proteins at the induced DSB sites were visualized. (B) Increase in the Venus fluorescence intensity at the laser-induced DSBs in the cells overexpressing olNbs1-Venus (No. 101, open circles, average in 4 cells; No. 301, open triangles, average in 4 cells) and in the cells expressing olNbs1 (H170)-Venus (No. 309, filled circles, average in 4 cells; No. 2202, filled triangles, average in 3 cells; No. 2502, filled squares, average in 6 cells) were measured after the micro-irradiation and plotted. The Y-axis represents fold increase in the fluorescence intensity determined by dividing by the mean intensity of the irradiated area of nuclei at 1.3 sec after the micro-irradiation. Time points are on the X-axis in sec. (C) Foci of olNbs1 or olNbs1 (H170) and γ-H2AX were visualized by immunostaining in the cells overexpressing olNbs1-Venus (No. 301) and in the cells expressing olNbs1 (H170)-Venus (No.309) at 2 and 12 h after the γ-ray irradiation (5 Gy). Foci in the not-irradiated cells are also shown (0 Gy). (D) Numbers of olNbs1 foci were counted at 30 min, 2, 6 and 12 h after the γ-ray irradiation (5 Gy) in the cells overexpressing olNbs1 -Venus (No. 301) and in the cells overexpressing olNbs1 (H170)-Venus (No. 309). The foci number in the not-irradiated cells were also counted (0 Gy). Bars represent standard errors of the averaged foci number per cell (n > 20 cells). Statistical analyses were performed using Student's *t* tests (*p < 0.01).

To validate whether the delay in DSB repair and the suppression of autophosphorylation of DNA-PKcs results from overexpression of olNbs1 (H170), we examined the accumulation of olNbs1 (H170)-Venus to DSB sites and its foci formation. The cells overexpressing olNbs1-Venus were micro-irradiated with a laser and DSBs were induced in the restricted regions of their nuclei. Recruitment of the both types of olNbs1-Venus to the laser-induced DSBs started to be observed from 16.3 s after the laser-irradiation and the intensity of the Venus signal reached a plateau at 211.3 s after the laser irradiation in both types of olNbs1. olNbs1 (H170)-Venus showed an increase in accumulation to the DSB sites at the plateau (Fig 4A and 4B).

We then examined the foci formation of olNbs1. Wild-type olNbs1 foci were increased 30 min after the γ-ray irradiation (5 Gy) and gradually decreased during the following 6 h (Fig 4C and 4D). In contrast, the number of olNbs1 (H170)-Venus foci increased from 30 min to 2 h after the irradiation, then decreased (Fig 4C and 4D). These results suggest that olNbs1 (H170) was retained to DSB site longer than Q170-type olNbs1.

### Molecular modeling prediction of tandem BRCT domains of olNbs1

We attributed elevated accumulation of olNbs1 (H170)-Venus to conformational change caused by Q170H amino acid mutation. Using the PYHRE2 program, Q170 of olNbs1 was predicted to locate on the surface of the protein and able to interact with another molecule with its side chain and the side chain of H170 was predicted to be on the surface of the BRCT1 domain (S5 Fig). From the previous report, NBS1 interacts with γ-H2AX through FHA-BRCT domain [2]. These observations suggest that Q170H mutation can change the molecular interaction between olNbs1 and the DSB repair scaffold proteins, such as γ-H2AX.

The phosphorylated tail of γ-H2AX binds to NBS1 through molecular contacts provided by both BRCT domains [23,24]. The five amino acid residues (V142, T143, K145, I147, L222, and K217) located in the groove between 2 tandem BRCT domains of olNbs1 and H170-type olNbs1 correspond to the residues of hNBS1 which are important for the molecular interaction between hNBS1 and the phosphorylated tail of γ-H2AX. The location of the Leu222 residue between olNbs1 and olNbs1 (H170) is predicted to slightly change, whereas 4 other residues would locate at the same position both in olNbs1 and H170-type olNbs1. This finding suggests that both olNbs1 and olNbs1 (H170) can interact with the phosphorylated tail of γ-H2AX.

R215 residue is known to form a salt bridge to D205 and E206 residues in the BRCT1 domain of hNBS1 conferring protein stability [25]. The side chain of R203 residue of olNbs1, which corresponds to R215 residue in hNBS1, is predicted to locate proximally to the side chain of E193 residue, suggesting that these residues can form a salt bridge to stabilize the globular structure of olNbs1. By contrast, the R203 residue of olNbs1 (H170) is predicted to distantly locate from E193 residue, suggesting that the globular stability of olNbs1 (H170) is lower than olNbs1.

## Discussion

In this study, we aimed to reveal the relationship between nonsynonymous polymorphisms in olNbs1 and DSB repair function using the variations within wild medaka populations. It is highly likely that olNbs1 Q170 residue has an important role in DSB repair from the following 2 findings: (1) olNbs1 Q170 residue corresponds to the Q185 residue of hNBS1, which has been reported to be associated with the increased risk of a variety of cancers (S2 Table); (2) the sequence around Q170 was highly conserved among animal species (Fig 1A). We found that olNbs1 H170 allele is specifically distributed in the eastern Korean group of medaka and further examined the impaired DSB repair in medaka cultured cells which overexpressed olNbs1 (H170)-Venus.

Cells over expressing olNbs1 (H170)-Venus showed delayed DSB repair (Fig 2) and suppressed phosphorylation of DNA-PKcs at T2609 (Fig 3). In mammalian cells, it is reported that DNA-PKcs is phosphorylated at T2609 by DNA-PK itself and also by the DSB repair related kinases such as ATM [26]. While both of the ATM inhibitor and DNA-PKcs inhibitor suppressed DNA-PKcs phosphorylation at T2609 in the medaka cells overexpressing wild-type olNbs1-Venus [15], only the ATM inhibitor did in the cells overexpressing olNbs1 (H170)-Venus (Fig 3), suggesting that autophosphorylation of DNA-PKcs is interfered by overexpression of olNBs1 (H170).

Our results showed that fluorescent signal at the DSB sites was stronger and increased more rapidly in the cells overexpressing olNbs1 (H170)-Venus than in the cells overexpressing wild-type olNbs1-Venus (Fig 4). This demonstrates that more olNbs1 (H170)-Venus protein accumulated for a longer period at the DSB sites than wild-type olNbs1-Venus. Since NBS1 is a component of MRN complex, these findings strongly suggest that recruitment of MRN complex to the DSB sites was enhanced in the cells overexpressing olNbs1 (H170)-Venus. MRN (MRX) complex can be a barrier to limit the accumulation of Ku heterodimer to DSB sites in yeast [27] and that interaction of ku80 and DNA-PKcs is prerequisite for recruitment of DNA-PKcs to DSB site [28]. These evidences imply a possible explanation of the suppressed autophosphorylation of DNA-PKcs in the cells overexpressing olNbs1 (H170)-Venus; The prolonged retention of MRN complex including the mutated olNbs1 might form a barrier for the resection of DSB ends in a dominant-negative manner, restrict the recruitment of Ku heterodimer to the DSB sites and decrease the activated DNA-PKcs that would be recruited to the DSB sites and autophosphorylated.

In our molecular prediction analysis, Q170H amino acid substitution caused the structural changes in the BRCT domains of olNbs1 (S5 Fig). Since the Mre11 binding domain of olNbs1 (A747—A758) is distant from H170 [29], it is likely that Mre11 binding domain of olNbs1 (H170) protein is intact and MRN complex is formed in the cells overexpressing olNbs1 (H170)-Venus. BRCT domains are essential for the physical interaction between NBS1 and γ-H2AX [2], the predicted conformational change in the BRCT domains of olNbs1 (H170) may increase the affinity of the BRCT domains to γ-H2AX, resulting the enhanced accumulation of olNbs1 (H170) at the DSB sites as shown in Fig 4, and the dominant-negative inhibition of DSB repair in the cells overexpressing olNbs1 (H170)-Venus. Thus, analysis of nonsynonymous polymorphisms of NBS1 in medaka wild populations provided us the facts to understand how NBS1 works and further study will be under taken to make clear the detailed molecular machinery of DSB repair.

Q170H mutation was specifically found in the eastern Korean group of medaka. Heterozygotes of olNbs1 (Q170/H170) were found in medaka originated from three locations (Yongcheon, Geoje and Gwangui) in the Korean peninsula, while homozygotes or heterozygotes were not observed in medaka originated from the 33 locations in the Japanese archipelago and the 3 locations in the eastern part of the Chinese continent (Shanghai, Kunming and Xuanlan in Table 1). Tajima's *D* showed the moderate significant negative value in the E.KOR group (S4 Fig). Our population genetic analysis suggests that the H170 allele is the descendant (S3 Fig) and the exon 5 of olNbs1, which contains Q170, has been positively selected in the populations of the E.KOR group (S2 Fig). These results suggest that Q170H amino acid substitution bestowed some evolutionary advantages for survival on the medaka in the eastern part of the Korean peninsula. Since NBS1 participates in repair process of spontaneous DSB repair by olNbs1 (H170) can increase the variation of immune cells and gametes in population [30,31]. In that case, olNbs1 (H170) allele can contribute to increase the probability of survival under rapid environmental changes, inducing the spread of olNbs1 (H170) allele in the E.KOR group. In our colony formation assay, the cells overexpressing olNbs1 (Q170)-Venus and the cells overexpressing olNbs1 (H170)-Venus showed the same viability against γ-rays (5–15 Gy) irradiation, strongly suggesting that olNbs1 (H170) allele do not make cells highly susceptible to irradiation (S6 Fig). The possible advantages for the survival of individuals with olNbs1 (H170) allele have to be elucidated by further studies.

In hNBS1, the allele frequency of Q185E differs among geographical populations (e.g. Q185 allele frequency in the 1000 genome database is 44.7% in Japanese in Tokyo and 30.8% in Toscani in Italy) [9]. Demogines et al. suggested that selection pressure was present on the Q185 residue of NBS1 during the evolution of primates, although the functional changes caused by Q185E amino acid substitution has not been addressed [10]. Our previous study demonstrated that medaka was a good model to provide an evolutionary significance of genetic variations in human because of analogous relationships between genetic polymorphisms and phenotypic traits in wild medaka populations to those in humans [17]. Therefore, human Q185E alleles might have govern the survival in a certain environment and spread across human populations as suggested by the possible evolutionary advantage of *olnbs1* in the wild medaka populations. Using variations within wild populations of medaka will provide a novel approach to explore the functional link between the polymorphisms in DNA repair genes and potential cancer risks in human.

## Materials and Methods

### Ethics

This research was conducted using protocols approved by the Institutional Animal Care and Use Committee of The University of Tokyo. The Approval (number: C14-02) was received prior to beginning research. All surgery on fish was performed using MS-222 and chilling as anesthesia, and all efforts were made to minimize suffering.

### *olnbs1* sequencing

Wild population of *Oryzias latipes* consists of 4 major groups genetically and geographically distinct: the northern Japanese (N.JPN), the southern Japanese (S.JPN), the east Korean (E.KOR), and the west Korean/Chinese (W.KOR) groups [32–34]. SNP information of inbred medaka strains (Hd-rR, HNI, Kaga, HSOK, Nilan) were obtained from the National BioResource Project (NBRP) medaka SNP browser (http://medaka.lab.nig.ac.jp/cgi-bin/gb2/gbrowse/medaka/).

We analyzed 56 individuals from 49 local sites (Table 1). Thirty-nine local populations of *O*. *latipes* were provided by Graduate School of Frontier Sciences, The University of Tokyo, which have been maintained for generations as closed colonies [35]. Ten local closed colony populations of *O*. *latipes* (Jindo, Geoje, Sokcho, Gwangui, Kunming, Xuanlan, Samsan, Guhang, Simcheon) were provided by NBRP medaka. *O*. *luzonensis* and *O*. *curvinotus* genomic DNA were supplied from NBRP and used as the out-group.

Medaka caudal fins were isolated from the fish lab-stocks and homogenized in a solution of 10% SDS, 0.5 M EDTA, and proteinase K (Wako, Osaka, Japan; used at the final concentration of 20 μg/mL). The fish were returned to fresh water after amputation. Total genomic DNA purified by phenol–chloroform extraction and subsequent isopropanol precipitation was used to amplify the 340 base pair (bp) sequence of *olnbs1* by polymerase chain reaction (PCR) using a pair of primers designed based on *olnbs1* sequence of Hd-rR (No. 60_olNBS_1_F, GTG ATC CAT CAG AAA CTT GTG GT, No. 33_R.Q170H_check, TCG TCG ATT TCA GGA TTG). To amplify the partial *olnbs1* sequence from the genomic DNA of the medaka lab-stock Jindo (the E.KOR group), *O*. *luzonensis* and *O*. *curvinotus*, another pair of primers were used (No. 157_F. Exon4, GTG GTT TGT TCA TCA TGC TTG GAC; No. 158_R1. Exon6, GGA ACA GTT GTT TAC GAG C).

The PCR products were purified by isopropanol precipitation and used as templates in direct sequencing reaction (following the protocol by provider), and were then analyzed using ABI3500 genetic analyzer (Thermo Fisher Scientific K.K., Yokohama, Japan). We determined the partial DNA sequences of *olnbs1* and compared 326 bp for the population genetic analysis as described below. These nucleotide sequences were deposited into the DNA database DDBJ/EMBL/GenBank (accession Nos. LC159330 –LC159391 which are listed in Table 1).

### Population genetic analysis

We excluded in/dels from each *olnbs1* sequence of wild medaka lab-stock, and then aligned them using MUSCLE [36] with a default setting in MEGA5 [37]. Subsequently, the *olnbs1* haplotypes were determined using PHASE version 2.1 [38] with default parameters implemented in DnaSP version 5 [39], then the haplotype-based *F*_ST_ (*G*_ST_) [40] for each exon and intron and Tajima’s *D* were calculated [41] by DnaSP version 5. To determine the evolutionary relationship between the *olnbs1* haplotypes, we constructed a median-joining network [42] using NETWORK 5 (http://www.fluxus-engineering.com/).

### Plasmid construction

The sequence of olNbs1 was amplified from first strand complementary DNA of Hd-rR embryos by using KOD-plus- polymerase (TOYOBO, Osaka, Japan) and primers designed based on the Hd-rR sequence of olNbs1 (NBS_shinF_xho1, CCGCTCGAGCCCGCTGTTTACATTTCTCG, NBS_shinR_linkerXho1, CCGCTCGAGACCTCCACCTCCCCTGAAC). The olNbs1 sequence was then subcloned into *Xho*I site of *pG-olphsp70*.*1-hRluc-Venus*, which enables transient and induced overexpression of olNbs1-Venus protein by heat induction at 41°C for 2 h [43]. To generate Q170H nonsynonymous mutation (CAG to CAT) the following primers were used, F.olNbs1_Q170H (CAACAGTGCTGTCCATCAAAAACGTCCGCC), and R.olNbs1_Q170H (GGCGGACGTTTTTGATGGACAGCACTGTTG). Overlapping PCR was conducted with KOD-plus-neo polymerase (TOYOBO), followed by digestion of the wild-type plasmid with *Dpn*I. Then, the amplicon was introduced into an XL10-Gold competent cell (Stratagene, Agilent Technologies, Santan Clara, CA, USA). The plasmid was verified by sequencing.

### Cell culture and transfection

A cell line Hd-rRe3 established from an embryo of wild-type medaka strain Hd-rR [44] were cultured in Leibovitz’s L-15 medium (L4386, Sigma-Aldrich, Merck KGaA, Darmstadt, Germany) supplemented with HEPES (10 mM, pH 7.5) and with 20% fetal bovine serum (Biological industries, Beit-Haemek, Israel). Hd-rRe3 cells were sustainably transfected with the olNbs1-Venus expression plasmids using a Neon electroporation system (Invitrogen Life Technologies Japan, Thermo Fisher Scientific K.K., Yokohama, Japan). We transfected 3 × 10^4^ cells with 2 μL of the plasmid (4–6 μg/μL) by electroporation under the following conditions: 1,300 V, 20 ms, for 2 pulses. Then, the cells were cultured overnight at 33°C, followed by selection with 1–5 μg/mL puromycin (Calbiochem, Merck KGaA, Darmstadt, Germany) for 2–3 days. Cell lines stably expressing olNbs1-Venus were then established after 10–14 days incubation and cloning procedures.

### γ-ray irradiation and chemicals

Cells on 35 mm glass-bottom dishes (D300110, Matsunami Glass, Tokyo, Japan) were exposed to various doses of γ-rays from a ^137^Cs source at a dose rate of 7.5 Gy/min (Elan 3000; MDS Nordion, Ottawa, Canada), within 6–18 h after olNbs1 overexpression induced by heat treatment (at 41°C for 2 h). ATM inhibitor, KU55933 (Calbiochem, Merck KGaA, Darmstadt, Germany) was dissolved in DMSO to prepare a 10 mM stock solution. DNA-PK inhibitor, NU7026 (Calbiochem, Merck KGaA) was dissolved in DMSO to prepare a 2 mM stock solution. Both chemicals are stored at -30°C. Before γ-ray irradiation (5 Gy), cells were incubated for 1 h in L-15 medium with the DNA-PK or ATM inhibitor at the final concentration of 10 μM.

### Laser micro-irradiation

Laser micro-irradiation analysis was performed as previously described with a few modifications [45,46]. We seeded 2 × 10^5^ of cultured cells expressing wild-type olNbs1-Venus or olNbs1 (H170)-Venus into the glass-bottom dishes and cultured the cells for 2 days at 33°C. After heat treatment (2 h) at 41°C to induce olNbs1-Venus or olNbs1 (H170)-Venus expression, the cells were incubated overnight and then incubated with 4 μM Hoechst 33258 (Wako, Osaka, Japan) in L15 medium supplemented with 20% FBS for 10 min at 33°C. Then, the cells were washed in L15 medium (phenol red free) before laser irradiation. Images were obtained using a SP6 confocal microscope (Leica Microsystems, Wetzlar, Germany) and analyzed using LAS AF software (Leica Microsystems). We set a strip-shaped region of interest (ROI) and 5 shots of 405 nm laser was irradiated into the ROI, inducing DSBs in the restricted part of the nuclei. Seventeen images were captured at 15 s intervals from 1.3 s to 241.3 s after the micro-irradiation, and then 3 more images were captured at 30 s intervals. The mean fluorescence intensity of the irradiated ROI in the nucleus was digitalized using Image J. Fold increase (F. I.) at each time point was calculated by following equation; F. I. (%) = 100 × ((mean intensity at each time point)–(mean intensity at t = 1.3 s))/(mean intensity at t = 1.3 s).

### Immunofluorescence staining

Immunofluorescence staining was performed as previously described [15, 47] with minor modifications. We seeded the cells (2 × 10^5^ cells per dish) in the 35 mm glass-bottom dishes and incubated overnight at 33°C before irradiation. After irradiation, the cells were incubated at 33°C for 0.5, 2, 6, or 12 h and then fixed by adding 0.5 mL of 4% paraformaldehyde in PBS per dish for 10 min at room temperature. After washing three times with PBS, the cells were permeabilized with 0.5% Triton X-100 in PBS for 30 min at 4°C and were incubated overnight with mouse monoclonal anti-phospho-H2AX (Ser139) antibody (Cat. No. 05–636, clone JBW301, Upstate, Millipore) or with mouse monoclonal anti-DNA-PKcs pT2609 antibody (Cat. ab18356, abcam) or with rabbit polyclonal anti-GFP antibody (MBL, Nagoya, Japan) at 1:5,000 dilution in blocking buffer containing 0.5% Triton X-100 and 0.2% normal goat serum at 4°C in a humidified box. After washed three times with PBS, the cells were incubated with Alexa Fluor 546 conjugated goat anti-mouse IgG (H + L) secondary antibody (A11003, Invitrogen) or with Alexa Fluor 488 conjugated goat anti-rabbit IgG (H + L) secondary antibody (A11008, Invitrogen) at 1:5000 in PBS containing 3% BSA for 1 h at room temperature in the dark. The cells were then washed three times with PBS and counterstained with 1 μg/mL 4′,6-diamino-2-phenylindole (DAPI, Wako) for 5 min at room temperature, washed with PBS, and finally immersed in 10% glycerol. Fluorescence images were obtained using a fluorescence microscope (IX-81, Olympus, Tokyo, Japan) equipped with a digital camera (DS Ri1, Nikon, Tokyo, Japan).

### Neutral comet assay

A neutral comet assay was performed as previously described with minor modifications [47]. We incubated 2 × 10^5^ cells in the glass-bottom dishes overnight at 33°C and then treated the cells with heat at 41°C for 2 h to induce Venus-tagged olNbs1. After the medium in the dishes was exchanged, the cells were further incubated for 14 h and then irradiated with γ-ray (5 Gy) as described. Thirty minutes after the irradiation, the cells were detached with 0.05% trypsin (Gibco-BRL) and 0.002% EDTA, and were suspended in L-15 medium. We mixed 30 μL of the cell suspension with 300 μL of 1% low-melting-temperature agarose (Lonza Japan, Tokyo, Japan) in PBS. The mixture was layered on top of a glass slide coated with 1% agarose and gently spread placing a coverslip on it. After putting the slide on ice for 3 min to solidify the agarose, the coverslip was gently removed, and the slide was immersed in a freshly prepared lysis buffer (2% SDS and 30 mM EDTA) for 30 min in the dark. After washing with TBE, electrophoresis was performed at 20 V for 25 min, and the agarose gel on the slide was subsequently soaked in 100 ng/mL propidium iodide for 5 min in the dark. At least 100 cells per slide were analyzed using a fluorescence microscope (BX-50, Olympus) equipped with a digital camera (DP-70, Olympus). Tail moment scores were calculated from the fluorescent image of each nuclei electrophoresed using Casp software [48].

### 3D modeling FHA-tandem BRCT domains of olNbs1

The amino acid sequences of FHA-tandem BRCT domains of wild-type olNbs1, olNbs1 (H170) and olNbs1 (SOK) proteins were applied to predict tertiary structural change of olNbs1 using PYHRE2 program [49]. Partial amino acid sequence of the N-termini of olNbs1 (between residues 22 and 311) was utilized for prediction. Models were based on the template of the *Saccharomyces pombe* NBS1 FHA/BRCT-repeat domain (PDB code: 3I0M).

### Statistical analysis

Student *t* tests were used to determine differences were significant when *p* < 0.01.

## Supporting Information

S1 Fig Amino acid sequence alignment of full-length olNbs1 (Hd-rR) and hNBS1. Red letters in olNbs1 represent polymorphic amino acid residues within the 5 inbred medaka strains. Closed triangles on hNBS1 represent disease-related residues; The mutation to valine at I171 residue is associated with higher (odds ratio 3.2) risk of breast cancer [9] and I171V allele frequency is 0.5% in Japanese population; Homozygote of Q185 is associated with higher (odds ratio 1.64–3.87) risk of a variety of cancers and leukemia (briefly reviewed in S2 Table), Q185 allele frequency is 44.7% in Japanese population; R215W mutation is found in NBS patients with a severe phenotype [50]. Blue, red, and green boxes represent FHA, BRCT1, and BRCT2 domains, respectively.
(TIF)

S2 Fig Haplotype-based *F*_ST_ (*G*_ST_) calculated for each exon and intron. Sequences from the 56 individuals were used to calculate haplotype-based *F*_ST_ (*G*_ST_) values using DnaSP version 5 [39,40].
(TIF)

S3 Fig Median-joining network of olNbs1 exon5. The circles and each color represent olNbs1 haplotypes (from H_1 to H_10) and the geographical groups of medaka (blue: N.JPN; orange: S.JPN; green: E.KOR; yellow: W.KOR; gray: sister species, *O*. *luzonensis and O*. *curvinotus*), respectively. The size of the circles indicates the frequency of the haplotypes and the nonsynonymous mutation for Q170H divides the H_6 haplotype group from the others. The red numbers on the branches indicate the positions of nucleotide differences on exon5 between the haplotypes.
(TIF)

S4 Fig Tajima’s *D* calculated based on 326 bp sequences. Tajima's *D* values were calculated based on the 326 bp (from exon 4 to intron 5 of *olnbs1*) for the 4 geographical groups of medaka. Sequences from the 56 individuals are used for calculation using DnaSP version 5 [39,41].
(TIF)

S5 Fig 3D modeling of olNbs1 (BRCT1-BRCT2 domain in N-terminus). Predicted structures of olNbs1 (Hd-rR) (A), olNbs1 (H170) (B), and olNbs1 (HSOK) (C) are drawn using PHYRE2 program [49] and PyMOL software (The PyMOL Molecular Graphics System, Version 1.8 Schrödinger, LLC.). Both Q170 and H170 residues are shown in the stick and ball model, which are positioned on the surface of the proteins. The 5 amino acid residues (V142, T143, K145, I147, L222 and K217) correspond to the residues interacts with which hNBS1 bind to the phosphorylated tail of γ-H2AX [23,24]. Glu193 and Arg203 are magnified in the parenthesized, those corresponds to the residues which would confer protein stability in hNBS1 [25].
(TIF)

S6 Fig Colony formation assay of cells overexpressing olNbs1-Venus (Nos. 101 and 301) or olNbs1 (H170)-Venus (Nos. 309 and 2202). The cells (2 × 10^5^) were seeded into the 35 mm glass-bottom dishes (D300110, Matsunami Glass, Tokyo, Japan) and cultured for overnight at 33°C. Sixteen hours after heat treatment (2 h) at 41°C, cells were exposed to 5, 10 and 15 Gy of γ-ray from a ^137^Cs source at a dose rate of 7.5 Gy/min (Elan 3000; MDS Nordion, Ottawa, Canada). After further cultured for 2 days, the cells were fixed by adding 0.5 mL of 4% paraformaldehyde in PBS per dish for 10 min at room temperature and stained with crystal violet to count the numbers of colonies. Survival rate of Hd-rRe3 cell line (not-transfected wild-type cells) were also shown by open rhombus. Three dishes were prepared in each dose for each cell line in one experiment and averaged survival rate of two independent experiments were shown.
(TIF)

S1 Table Classification of 40 nonsynonymous mutations of olNbs1 among 5 inbred strains of medaka. Forty nonsynonymous SNPs were found among 5 inbred strains (Hd-rR, HNI, Kaga, HSOK and Nilan) in this study and can be classified into 5 groups. Among them, 4 SNP groups are each specific to the 4 geographical groups of medaka [20,21]. Two SNPs which give amino acid substitutions of G542S and I695T are shared among the geographical groups and no SNP other than these are shared among the geographical groups of medaka. This finding strongly suggests that each SNP except for G542S and I695T appeared after the differentiation of each geographical group. It is also suggested that geographical groups of medaka can be classified into 2 large groups: a group of the southern Japanese group and the northern Japanese group and the other group of the eastern Korean group and the China-western Korean group. * represents Q170H in the HSOK specific amino acid substitutions.
(XLSX)

S2 Table Association of Q185E polymorphism and cancer risk in human. Homozygote of Q185 allele is associated with higher risks of a wide variety of cancer and leukemia. On the other hand, some studies have reported that E185 allele increases cancer risk in the cases of lung cancer in Chinese local population [51], basal cell carcinoma in Hungary, Romania and Slovakia [52]. These studies suggest that the amino acid substitution at Q185 residue in hNBS1 has some biological significance and that the impact of Q185E mutation can be two-sided depending on the environments. CI, confidence interval.
(XLSX)
