# Supplementary figures and images for: An Approach to Elucidate NBS1 Function in DNA Repair Using Frequent Nonsynonymous Polymorphism in Wild Medaka (Oryzias latipes) Populations
Source: PLoS One. 2017 Jan 20;12(1):e0170006. doi: 10.1371/journal.pone.0170006 (PMC5249114; doi:10.1371/journal.pone.0170006)

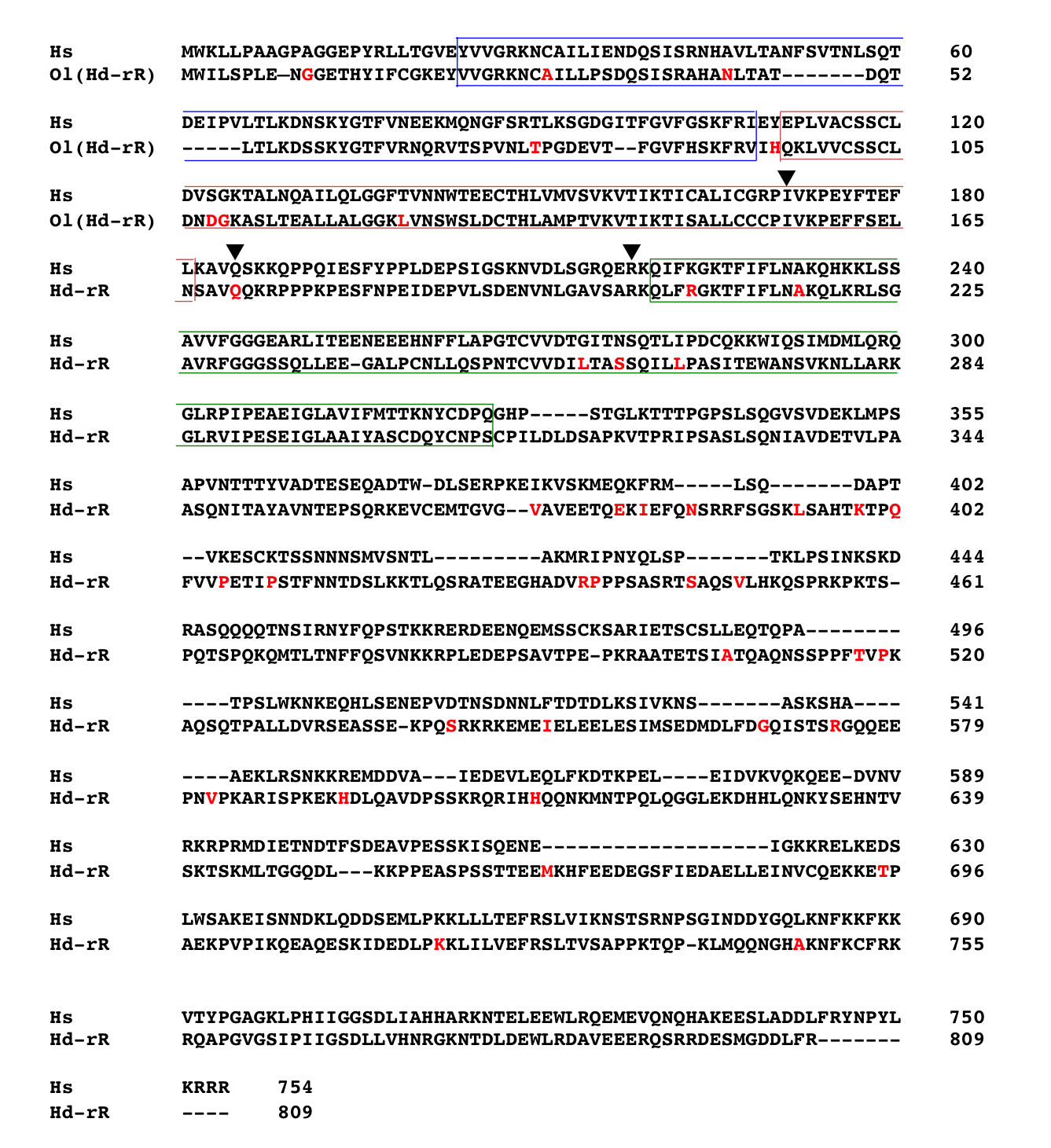

Supplement: S1 Fig — Red letters in olNbs1 represent polymorphic amino acid residues within the 5 inbred medaka strains. Closed triangles on hNBS1 represent disease-related residues; The mutation to valine at I171 residue is associated with higher (odds ratio 3.2) risk of breast cancer [9] and I171V allele frequency is 0.5% in Japanese population; Homozygote of Q185 is associated with higher (odds ratio 1.64–3.87) risk of a variety of cancers and leukemia (briefly reviewed in S2 Table), Q185 allele frequency is 44.7% in Japanese population; R215W mutation is found in NBS patients with a severe phenotype [50]. Blue, red, and green boxes represent FHA, BRCT1, and BRCT2 domains, respectively. (TIF) [file pone.0170006.s001.tif]

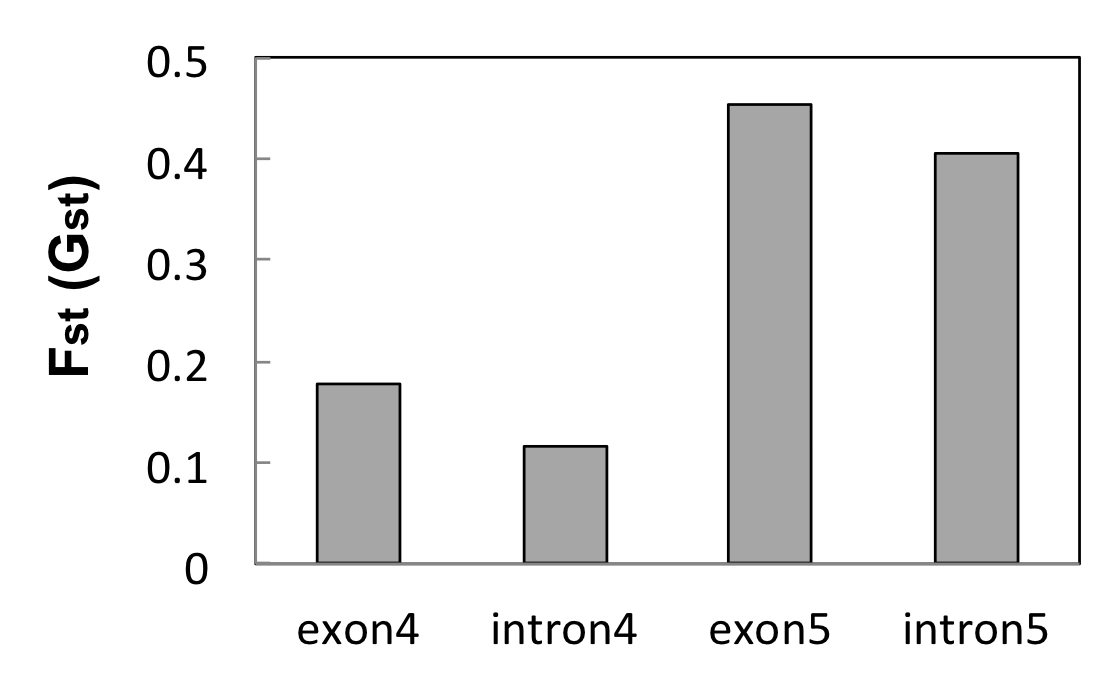

Supplement: S2 Fig — Sequences from the 56 individuals were used to calculate haplotype-based FST (GST) values using DnaSP version 5 [39,40]. (TIF) [file pone.0170006.s002.tif]

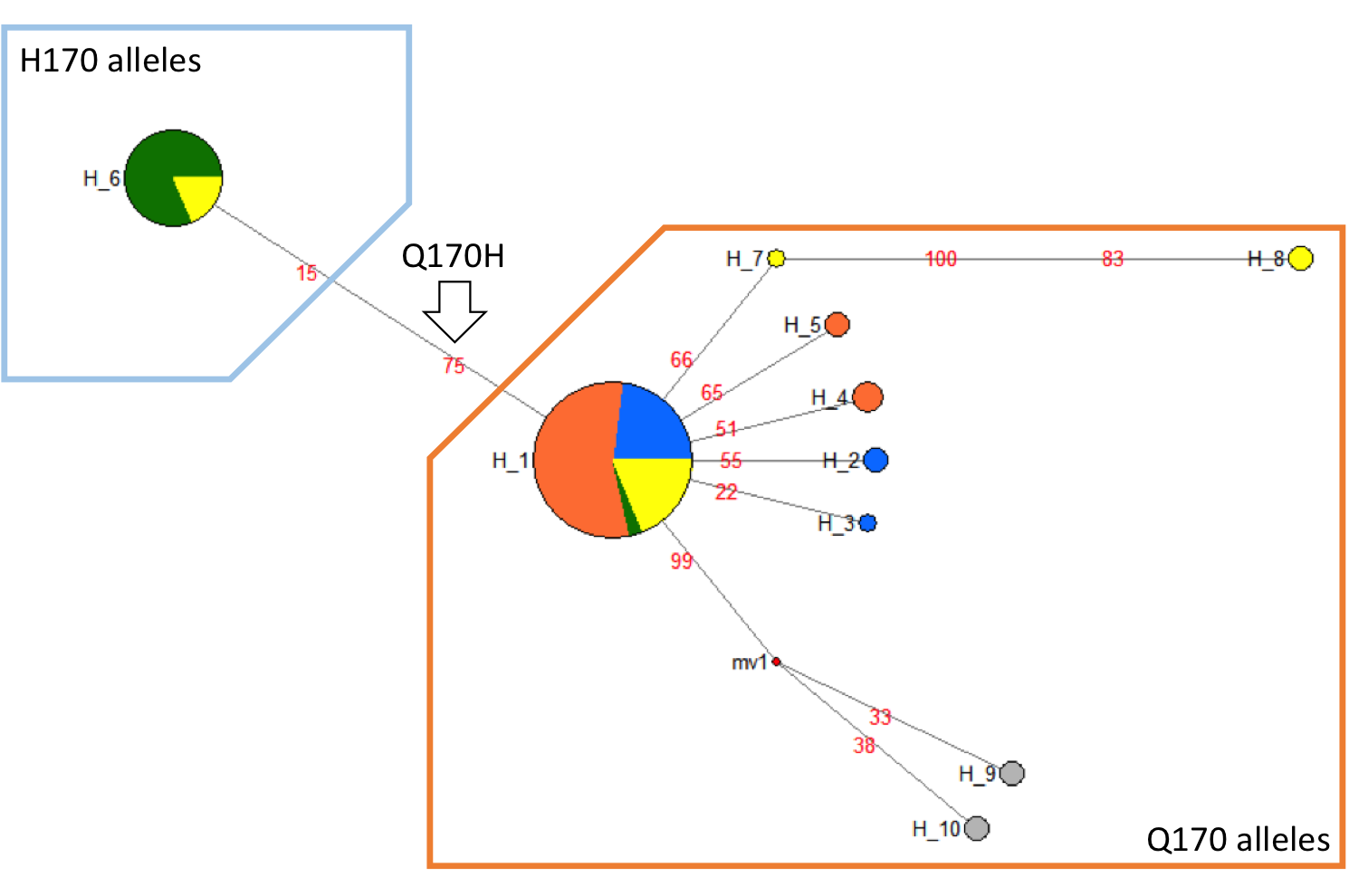

Supplement: S3 Fig — The circles and each color represent olNbs1 haplotypes (from H_1 to H_10) and the geographical groups of medaka (blue: N.JPN; orange: S.JPN; green: E.KOR; yellow: W.KOR; gray: sister species, O. luzonensis and O. curvinotus), respectively. The size of the circles indicates the frequency of the haplotypes and the nonsynonymous mutation for Q170H divides the H_6 haplotype group from the others. The red numbers on the branches indicate the positions of nucleotide differences on exon5 between the haplotypes. (TIF) [file pone.0170006.s003.tif]

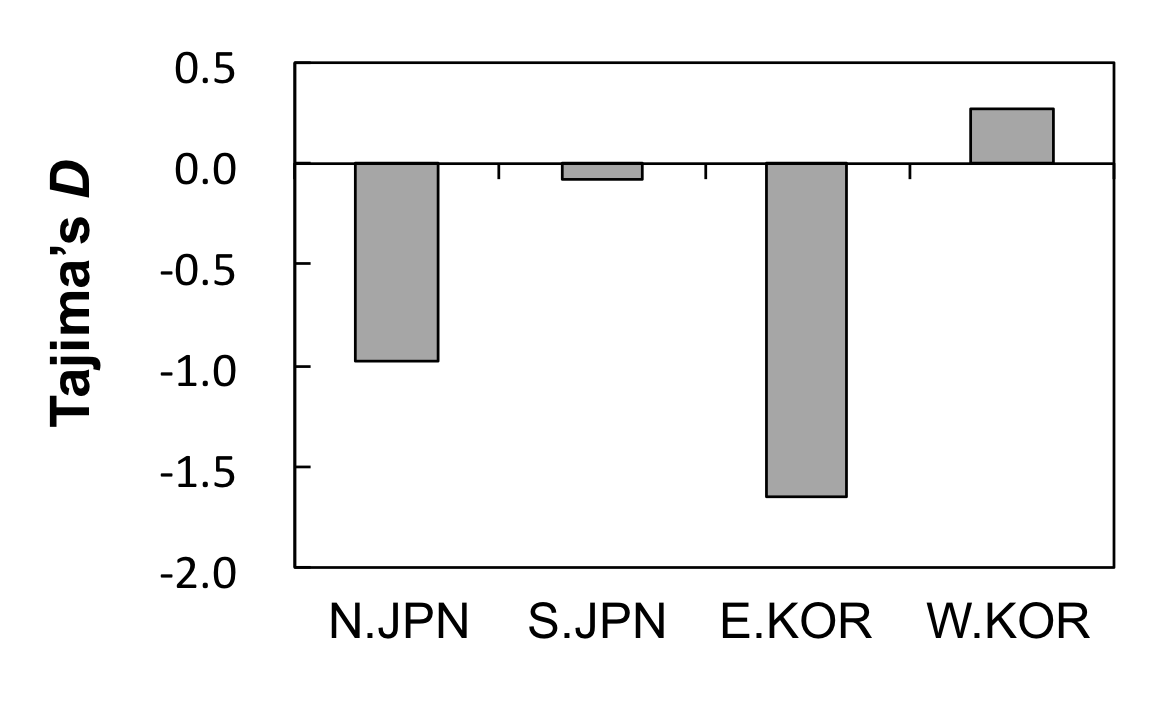

Supplement: S4 Fig — Tajima's D values were calculated based on the 326 bp (from exon 4 to intron 5 of olnbs1) for the 4 geographical groups of medaka. Sequences from the 56 individuals are used for calculation using DnaSP version 5 [39,41]. (TIF) [file pone.0170006.s004.tif]

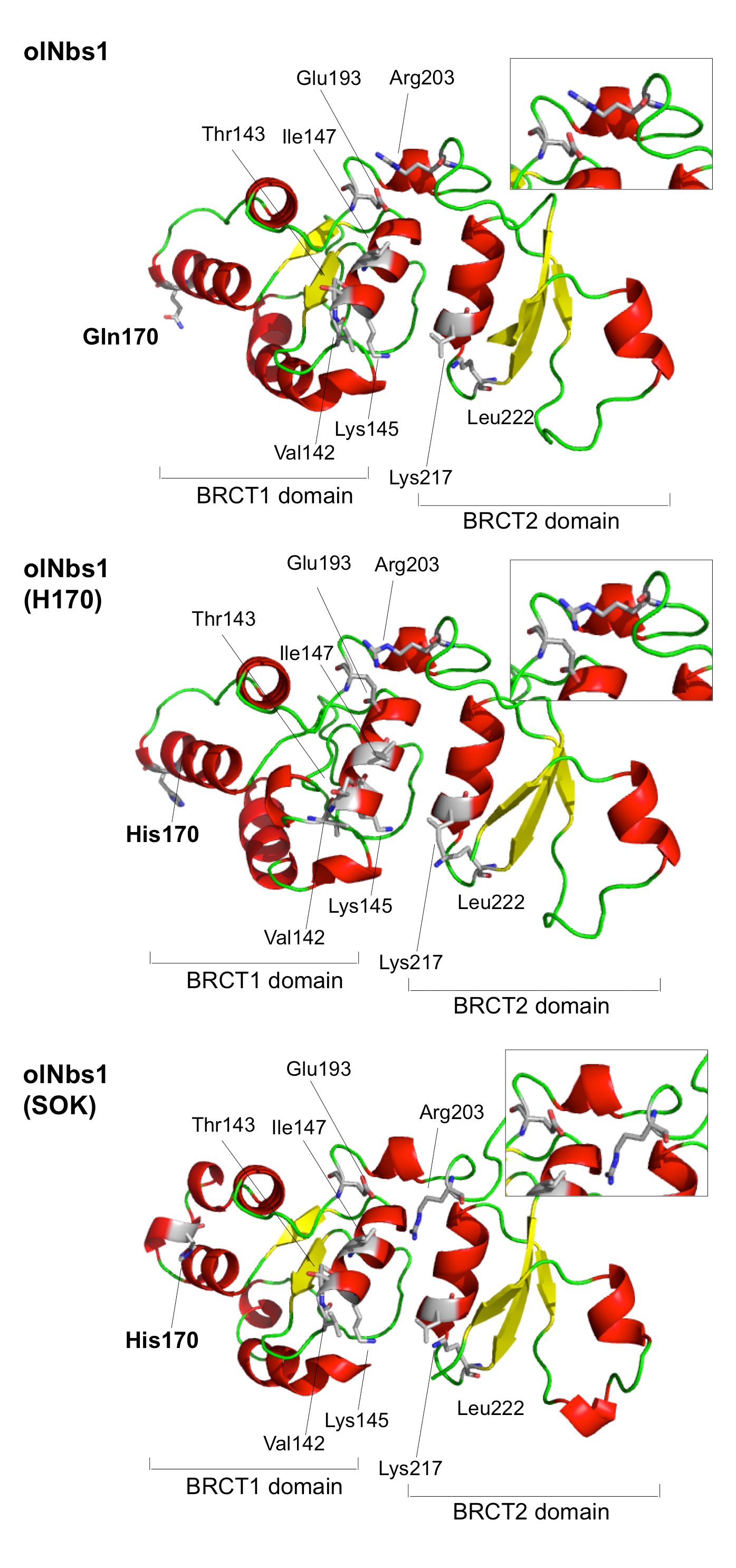

Supplement: S5 Fig — Predicted structures of olNbs1 (Hd-rR) (A), olNbs1 (H170) (B), and olNbs1 (HSOK) (C) are drawn using PHYRE2 program [49] and PyMOL software (The PyMOL Molecular Graphics System, Version 1.8 Schrödinger, LLC.). Both Q170 and H170 residues are shown in the stick and ball model, which are positioned on the surface of the proteins. The 5 amino acid residues (V142, T143, K145, I147, L222 and K217) correspond to the residues interacts with which hNBS1 bind to the phosphorylated tail of γ-H2AX [23,24]. Glu193 and Arg203 are magnified in the parenthesized, those corresponds to the residues which would confer protein stability in hNBS1 [25]. (TIF) [file pone.0170006.s005.tif]

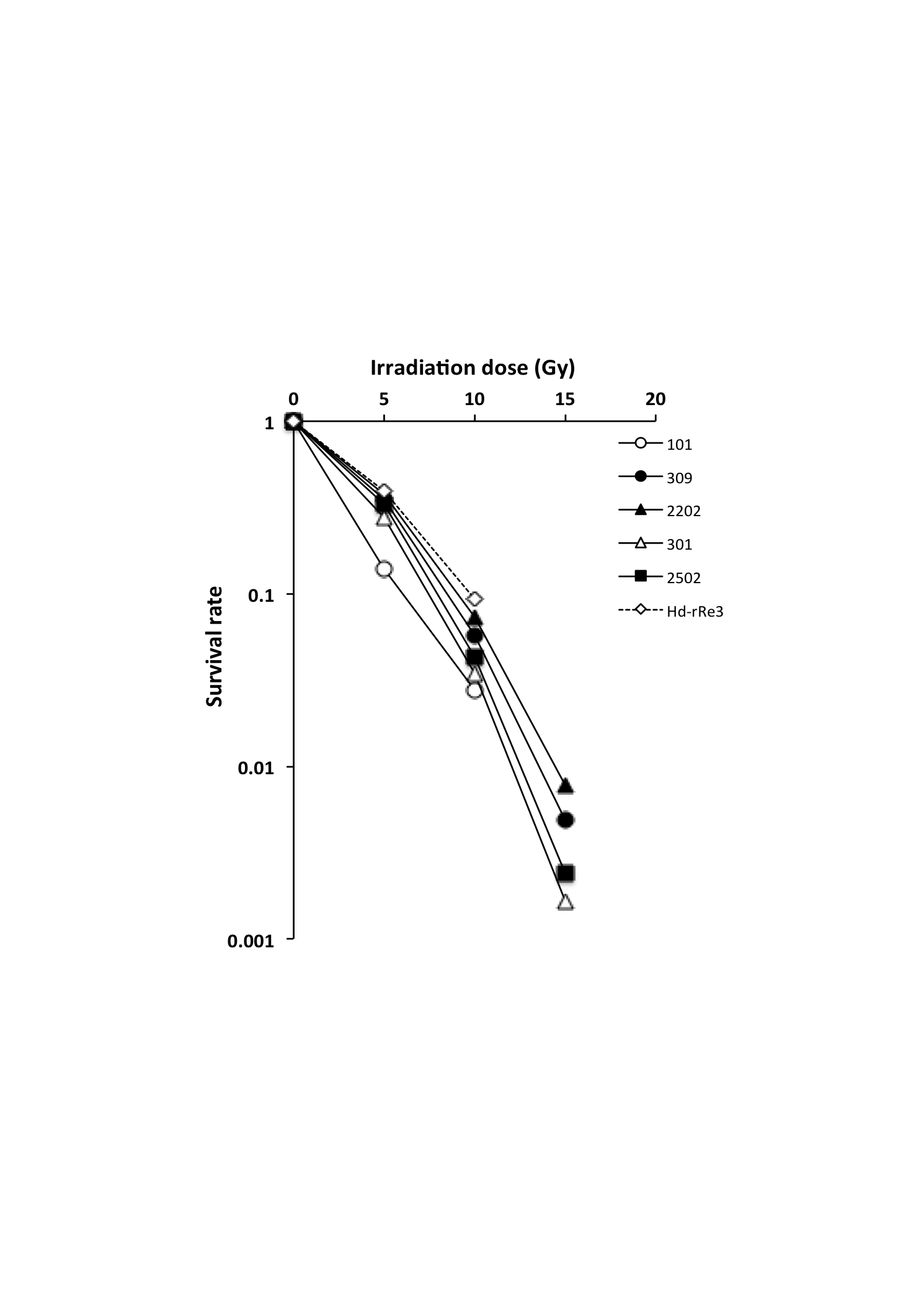

Supplement: S6 Fig — The cells (2 × 10^5^) were seeded into the 35 mm glass-bottom dishes (D300110, Matsunami Glass, Tokyo, Japan) and cultured for overnight at 33°C. Sixteen hours after heat treatment (2 h) at 41°C, cells were exposed to 5, 10 and 15 Gy of γ-ray from a 137Cs source at a dose rate of 7.5 Gy/min (Elan 3000; MDS Nordion, Ottawa, Canada). After further cultured for 2 days, the cells were fixed by adding 0.5 mL of 4% paraformaldehyde in PBS per dish for 10 min at room temperature and stained with crystal violet to count the numbers of colonies. Survival rate of Hd-rRe3 cell line (not-transfected wild-type cells) were also shown by open rhombus. Three dishes were prepared in each dose for each cell line in one experiment and averaged survival rate of two independent experiments were shown. (TIF) [file pone.0170006.s006.tif]
